# Proteomic and metabolomic studies on chilling injury in peach and nectarine

**DOI:** 10.3389/fpls.2022.958312

**Published:** 2022-10-04

**Authors:** Susan Lurie

**Affiliations:** Department of Postharvest Science, Volcani Center, Agricultural Research Organization, Rishon LeZion, Israel

**Keywords:** *Prunus persica*, mealiness, wooliness, internal reddening, internal browning

## Abstract

Peaches and nectarines are temperate climate stone fruits, which should be stored at 0°C to prevent the ripening of these climacteric fruits. However, if stored for too long or if stored at a higher temperature (4 or 5°C), they develop chilling injury. Chilling injury damage includes (1) dry, mealy, wooly (lack of juice) fruits, (2) hard-textured fruits with no juice (leatheriness), (3) flesh browning, and (4) flesh bleeding or internal reddening. There are genetic components to these disorders in that early season fruits are generally more resistant than late season fruits, and white-fleshed fruits are more susceptible to internal browning than yellow-fleshed fruits. A recent review covered the recent research in genomic and transcriptomic studies, and this review examines findings from proteomic and metabolomics studies. Proteomic studies found that the ethylene synthesis proteins are decreased in cold compromised fruits, and this affects the processes initiated by ethylene including cell wall and volatile changes. Enzymes in metabolic pathways were both higher and lower in abundance in CI fruits, an indication of an imbalance in energy production. Stress proteins increased in both fruits with or without CI, but were higher in damaged fruits. Metabolomics showed the role of levels of sugars, sucrose, raffinose, galactinol, and glucose-6-phosphate in protection against chilling injury, along with other membrane stabilizers such as polyamines. Amino acid changes were inconsistent among the studies. Lipid species changes during storage could be correlated with sensitivity or resistance to CI, but more studies are needed.

## Introduction

Peach (*Prunus persica* (L) Batsch) and nectarine (*P. persica* (L), var. *nectarina*) are stone fruits. They have a lignified endocarp (pit or stone) that encloses the seed, a fleshy mesocarp, and a thin exocarp. Nectarine cells have smaller intercellular spaces than peaches and are, therefore, denser. In addition, they lack pubescence on the skin, which is controlled by a single gene (Lill et al., 1989). On the basis of the separation of the stone from the flesh, peaches and nectarines can be divided into two groups; freestone and clingstone. In addition, based on the softening of the flesh that occurs during ripening, there are melting and non-melting types. Melting fruits will soften to < 8 N, while non-melting fruits will not. There are also white-fleshed and yellow-fleshed cultivars. The white-fleshed fruits tend to be lower in acidity and therefore taste sweeter, while the yellow-fleshed fruits have a more sweet–sour taste.

Peach and nectarine are climacteric fruits and will ripen both on and off the tree. At normal summer temperatures, they will soften and decay rather quickly, and cold storage is utilized to slow the ripening process and deter decay. However, chilling injury (CI) limits the storage life of peach and nectarine under low temperatures. CI develops during ripening at 20°C after the fruits are stored from 2 to 6°C for 2 weeks or longer or stored at 0°C for 3 weeks or longer (Lurie and Crisosto, 2005). The development of CI is internal and not visible until the fruits are cut open. CI manifests itself as (1) dry, mealy, wooly (lack of juice) fruits, (2) hard-textured fruits with no juice (leatheriness), (3) flesh browning, and (4) flesh bleeding or internal reddening (Lurie and Crisosto, 2005). Browning is often seen in mealy or leathery fruits, although it may occur in the absence of mealiness/wooliness, when enzymes such as polyphenol oxidase (PPO) act on phenolic substrates (Crisosto et al., 1999; Peace et al., 2006). Flesh bleeding is the spread of red pigment, anthocyanins, through the fruits' flesh during cold storage or after subsequent ripening (Peace et al., 2006). Mealiness/wooliness and leatheriness are the fruits' flesh textural disorders where affected ripe fruits have a dry grainy feel when chewed. In simple terms, mealy/wooly fruits are dry and soft when ripe, whereas leathery fruits have a dry and firm texture when ripe. CI is genetically influenced and triggered by a combination of storage temperature and storage period.

Mealiness/wooliness is a texture disorder shown as a lack of juice as the fruit softens and ripens. It is determined both visually after halving the fruits, and by measuring the expressible juice from the fruits (Lurie and Crisosto, 2005). The phenomenon has been associated with an imbalance in the activities of cell wall degrading enzymes due to an accumulation of de-methyl esterified pectins that are not depolymerized (Lurie et al., 2003; Lauxmann et al., 2012). Pectins form a gel structure that captures free water from the flesh, resulting in a mealy or wooly phenotype (Zhou et al., 2000). Cell wall enzymes such as pectin methylesterase (PE), polygalacturonase (PG), endo-1,4-glucanase (endoGL), and expansin (EXP) have been reported to be related to mealiness (Obenland et al., 2003; Lurie and Crisosto, 2005; Fruk et al., 2014). In addition, there may be a relationship between maturity date and mealiness/wooliness, since, in general, early-season cultivars are less susceptible than late-season cultivars (Lurie and Crisosto, 2005; Crisosto and Valero, 2008).

The other major chilling injury symptom is flesh browning, which is more prominent among the white-fleshed peaches and nectarines. The direct factors responsible for internal browning are phenolic substrates, browning enzymes, and enzymes acting on these substrates including polyphenol oxidase (PPO) and peroxidase (POD). Changes in membrane permeability in cold storage allow for the substrates to come into contact.

There are a number of interventions that can be utilized to delay the development of CI, some only experimental and others in limited commercial usage. Temperature manipulations are one approach, either delayed storage (low-temperature conditioning) or intermittent warming during the storage period. A controlled or modified atmosphere is also beneficial. In addition, there are physical stresses and chemical treatments which have been shown to induce processes that enhance resistance to CI. These include UV, heat treatment, salicylic acid, jasmonic acid, glycine betaine, and nitrous oxide. Lastly, there are hormonal interventions, particularly preharvest sprays with gibberellin and treatment with ethylene during storage. A review in 2005 discussed the biological basis of the different CI appearances and approaches to delay the appearance of chilling injury, both orchard practices and post-harvest manipulations (Lurie and Crisosto, 2005). These have been updated in recent reviews (Fruk et al., 2014; Rodrigues et al., 2020). A further update of the Lurie and Crisosto review focused on genomic and transcriptomic studies which endeavored to find quantitative trait loci (QTLs), genes, and gene regulons responsible for chilling injury sensitivity or resistance (Lurie, 2021). The present review will examine proteomic and metabolomic studies of chilling injury.

## Proteomics

A limitation of studies of transcriptomic changes in fruits is an inability to reveal post-translational modifications, which can be studied using proteomics. A recent review of proteomic profiling of horticultural commodities in post-harvest looked at the uses of this method (Mathabe et al., 2020). They include the identification of biomarkers for quality and for abiotic stress (temperature, controlled atmosphere); using protein markers during post-harvest handling; protein markers for breeding purposes; protein markers for the early identification of physiological and pathological disorders. These are the result of proteomic studies called translational plant proteomics which is aimed at predicting outcomes of practices involving plants (Agrawal et al., 2012). The word translational indicates that a proteomic study can be translated from basic analysis to an application such as food quality, food safety assessment, or food nutritional assessment (Agrawal et al., 2012).

**Figure 1:**
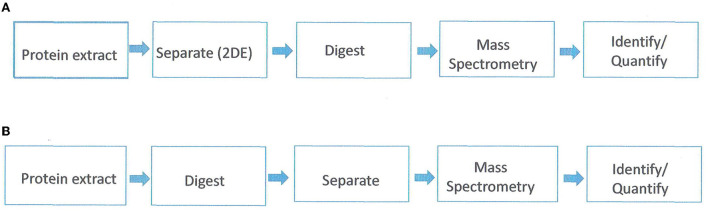
Proteomics/mass spectrometric workflow. **(A)** gel-based approach. **(B)** gel-free approach. Adapted from Petreschi et al. (2010).

Proteomics is the study of the whole set of proteins encoded by a genome. It addresses three biological aspects; protein expression, protein structure, and protein function. Proteomics studies can be with either gel-based or gel-free separation of proteins in conjunction with mass spectrometry (MS) and bioinformatics (Figure 1). The gel-based proteomics approach relies on two-dimensional electrophoresis (2-DE) for the separation of proteins based on their isoelectric point and molecular weight. The spots are then eluted from the gel and analyzed with mass spectroscopy (MS). One of the limitations of 2-DE is a high gel-to-gel variation which makes it difficult to distinguish biological from experimental variation. To overcome this, a two-dimensional in-gel electrophoresis (DIGE) was developed (Unlu et al., 1997). In DIGE, the samples are labeled prior to electrophoretic separation with dyes (Cy2, Cy3, and Cy5). Subsequently, the samples are mixed prior to isoelectric separation and then resolved together on the same 2-DE gel. Gel-based proteomics is the most powerful option for non-model plants (i.e., not Arabidopsis or rice). However, limitations are a bias toward highly abundant proteins, non-resolution of hydrophobic or very acidic proteins, and co-migration of proteins resulting in spots containing multiple proteins (Carpentier et al., 2008).

The gel-free approach first proteolyzes the mix of proteins into peptides which are then separated based on hydrophobicity *via* reverse-phase chromatography. Subsequently, the eluted peptides are analyzed with MS and the tandem mass spectra are then used to search databases and reconstruct the original proteins (Roe and Griffin, 2006). This approach is successful for relatively simple protein mixtures from sequenced species, although complex samples can be analyzed by using multidimensional protein identification technology (MudPit) (Washburn et al., 2001). With the gel-free approach, membrane proteins and low abundant proteins can be analyzed. However, gel-free methods cannot determine qualitative and quantitative protein isoforms or differential post-translation modifications (Carpentier et al., 2008).

### Proteomics of ripening

Knowing the changes in the proteome that occur during ripening can help to understand the changes occurring during CI. Many changes occur during the ripening of peaches and nectarines and these are reflected in changes in the proteasome. Since fruits are climacteric, there is an increase in the respiration rate, concomitant with the enhancement of ethylene synthesis. Together with the action of other plant hormones, the ethylene increase is the main signal for the initiation and control of fruit ripening. The changes during ripening include metabolic changes in acids and sugars, peel and mesocarp color changes, development of aroma compounds, and flesh softening. These changes occur only partially if fruits are stored for too long and develop CI.

There are a number of studies that compare ripening among different cultivars (Prinsi et al., 2011; Nilo et al., 2012; Jiang et al., 2020a). Other studies treated fruits to inhibit (1-methylcyclopropene [1-MCP], heat, chlorogenic acid) or advance ripening (ethephon), and examined changes in the proteome (Lara et al., 2009; Zhang et al., 2012; Jiang et al., 2014; Xi et al., 2017).

Nilo et al. (2012) conducted a global evaluation of the mesocarp proteome using 2-DE comparing firm and soft fruits from five peach and nectarine cultivars in order to see the common patterns of changes during ripening. Of the 271 differentially accumulated spots, 164 changed their abundance during softening. There were some spots that changed only in one or two cultivars and some which changed in all five. An average of 15% of the proteins changed between the firm and soft fruits in the five cultivars, which is close to the 17% found in a transcriptomic study of “Fantasia” nectarines (Vizoso et al., 2009). However, in the transcriptomic study, the number of transcripts increased during softening, while in the proteomic study, there were fewer proteins in soft fruits. All five cultivars examined by Nilo et al. (2012) were melting varieties. Prinsi et al. (2011) examined ripening in two cultivars with radically different softening patterns, non-melting “Oro A” and melting “Bolero.” Using 2-DE, they found that 53 spots changed significantly in relation to the ripening stage and genetic background.

Another study compared two cultivars of peaches that undergo limited flesh softening, stony-hard “Xiacui,” and a hard melting flesh “Xiahui 8” (Jiang et al., 2020a). The stony-hard cultivars produce very little ethylene during fruit ripening (Hayama et al., 2006). Using iTRAQ labeling and gel-free fractionation and analysis, they detected about 4,000 proteins in each cultivar. There were 57 proteins in the stony-hard cultivar and 190 proteins in the hard melting flesh cultivar that showed more than a 1.5-fold abundance change between harvest and ripening (15 days at 25°C for stony-hard and 6 days for hard-melting flesh). Jiang et al. (2020b) also examined the hard melting flesh “Xiahui 8” proteome using iTRAQ from 7 days before harvest to 8 days at 25°C. They found 387 proteins with more than 1.2-fold difference and 141 with more than 1.5 differences in abundance. In addition, Wu et al. (2016) purified mitochondria from “Xiahui 8” fruits at different times during storage at either 25°C or 4°C and compared their proteomes. There were 24 differentially expressed proteins (DEPs) (2-fold) out of 300 spots that were identified.

The studies on treatments to inhibit or enhance ripening all used 2-DE and found 47 DEPs between control fruits and those treated with hot water (48°C for 10 min) and 1-MCP (Jiang et al., 2020b), 57 DEPs when fruits were hot air heated 3 days at 39°C (Lara et al., 2009), 38 DEPs when fruits were treated with 1-MCP or ethephon (Zhang et al., 2012), and 18 DEPs when fruits were treated with chlorogenic acid (Xi et al., 2017).

#### Hormones

Peaches and nectarines are characterized by a climacteric increase in ethylene during ripening. Nilo et al. (2012) found that two 1-aminocyclopropane-1-carboxylic acid oxidase (ACO) spots increased in soft fruits of five melting flesh cultivars along with several other proteins that increased in soft fruits and have been reported to be transcriptionally regulated by ethylene (Hayama et al., 2006), including endo-PG and EXP which also increased. Prinsi et al. (2011) comparing melting and non-melting peaches during ripening found that ACO had one of the largest increases in abundance, but changes in other ethylene metabolism proteins, S-adenosylmethionine synthase (SAM synthase) and β-cyanoalanine synthase (β-CAS) were also seen. Two forms of ACO were found, both increased during ripening, but one form increased 19-fold in the melting flesh “Oro A,” and 2.5-fold in the non-melting “Bolero.” SAM synthase was higher in abundance in the melting flesh cultivar, although both this enzyme and β-CAS decreased in both fruits between the unripe and ripe stages. Lara et al. (2009) reversibly inhibited the ripening of peaches by hot air treatment and observed inhibition of SAM. Zhang et al. (2012) induced ACO with the ethephon treatment, while 1-MCP repressed it. To examine the different enzymes involved in the SAM cycle and ethylene biosynthesis which lead to ripening, Zeng et al. (2020) generated a monoclonal antibody library from a melting flesh peach and a stony-hard peach and used it to screen peach fruit proteins. They found 1,587 DEPs and 33 were identified by immunoprecipitation and MS analysis. In addition to the three enzymes in the ethylene pathway, SAM synthase, ACS, and ACO, they identified the enzymes synthesizing methionine as important in regulating ethylene production.

Jiang et al. (2020b) found that an enzyme in the abscisic acid (ABA) pathway, 9-cis-epoxycarotenoid dioxygenase (NCED), was induced at harvest and had a higher abundance from harvest to the end of shelf life. An abscisic acid ripening-like protein (ACS) was found in ripe peaches (Prinsi et al., 2011) and in fruits treated with ethephon (Zhang et al., 2012), and absent in peaches treated with heat and 1-MCP to inhibit ripening (Jiang et al., 2014), or 1-MCP alone (Zhang et al., 2012).

#### Cell wall enzymes

Endo-PG and EXP were found in the ripe peaches and nectarines in higher abundance than the unripe fruits (Nilo et al., 2012). Endo-PG also increased in the hard-melting flesh during ripening but not in the stony-hard cultivar (Jiang et al., 2020a). Jiang et al. (2020b) followed ripening of the hard-melting flesh “Xiahui 8”, and found endo-PG, pectase lyase (PL) and pectinesterase (PE) significantly up-regulated during shelf life up to d 6 and β-mannosidase (MN), a hemicellulose hydrolyze, over 2-fold higher compared to harvest. Pectin esterase inhibitor (PEI) was found in peaches treated with heat to inhibit ripening and might affect softening, by preventing PE activity and thus the access of endo-PG to cell wall pectin (Lara et al., 2009; Jiang et al., 2014).

In addition, two PL proteins were found in the hard melting flesh and only one was present in the stony-hard cultivar (Jiang et al., 2020a,b). Also, two enzymes related to lignin synthesis were found, cinnamyl alcohol dehydrogenase (CAD) in non-melting fruits (Prinsi et al., 2011), and cinnamoyl CoA reductase (CCR) in both the hard melting flesh and stony-hard fruits (Jiang et al., 2020a). The authors speculate that the lignin pathway may play a role in maintaining flesh firmness, as has been suggested for loquat fruits (Cai et al., 2006).

#### Sugar and organic acid metabolism

During ripening, sugar levels increase and organic acids decrease in peaches and nectarines. Along with fructose and glucose, sucrose is one of the main carbohydrates in peach fruits, and during ripening, it decreases while glucose and fructose levels increase. Enzymes controlling sucrose levels include sucrose synthase and neutral invertase (NI). Nilo et al. (2012) found a neutral invertase inhibitor (INH) in all five cultivars of melting flesh fruits which increased in ripe fruits. Lara et al. (2009) held peaches for 3 days at 39°C and then in 20°C shelf life. The heat treatment reversibly inhibited ripening. Acid invertase (AI) was totally inhibited, while NI was less affected.

In the hard melting flesh fruits, there was an increase in sucrose phosphate synthase (SPS) protein (Jiang et al., 2020a), while a soluble sucrose synthase (SUSY) was found to increase during ripening (Prinsi et al., 2011). Both enzymes are involved in sucrose import.

In “Xiahui 8” during ripening, DEPs related to starch and sucrose metabolism and pentose phosphate pathway (such as trehalose-phosphate synthase (TPS), beta-glucosidase (β-GC), glucose-6-phosphate 1-dehydrogenase (G6PDH) and phosphofructokinase (PFK), were all higher at the end of ripening (Jiang et al., 2014). Nilo et al. (2012) found two proteins from the oxidative pentose phosphate pathway, G6PDH, and 6-phosphogluconate dehydrogenase (6-PGD). 6-PDG increased during ripening, while G6PDH either remained constant or decreased in the five cultivars. Both these enzymes generate NADPH and another enzyme that generates NADPH and which increased in the ripe fruit was NADP isocitrate dehydrogenase (NADP-ISO). A NADP-dependent malic enzyme (NDME) breaks down malic acid to pyruvate and NADP and increases in the hard-melting flesh fruits (Jiang et al., 2020a). The generation of NADPH can help maintain the redox potential in the cells.

The predominant organic acids in ripe peaches are malic and citric acids, which are substrates for the generation of oxalacetate (OAA). OAA can in turn be used to synthesize phosphoenolpyruvate (PEP), through phosphoenolpyruvate carboxykinase (PCK). Nilo et al. (2012) found three PCK spots in all the melting flesh cultivars which increased during ripening. The melting flesh cultivar in Prinsi et al. (2011) also showed an increase in NAD-dependent malate dehydrogenase (NAD-MDH) which catalyzes the reversible reaction between OAA and malic acid, while the non-melting cultivar did not have this protein spot. In “Xiahui 8,” succinate dehydrogenase (SDH), an enzyme involved in both the citric acid cycle and electron transport chain was highest at harvest (Jiang et al., 2020b). In heated peaches, PCK increased after treatment, suggesting that under heat stress organic acid respiration is preferred over sugar metabolism (Lara et al., 2009).

#### Organelles

Ribulose-1,5-bisphosphate carboxylase/oxygenase (Rubisco) is involved in the first step of carbon fixation in photosynthesis and is a very abundant protein. It decreased during fruit ripening (Jiang et al., 2020a,b). Prinsi et al. (2011) found a 0.5-fold decrease of Rubisco in the non-melting flesh peach during ripening and a 3-fold decrease in the melting peach cultivar.

Ferredoxin-NADP reductase and oxygen-evolving enhancer protein 2, part of the PSII complex in chloroplasts, were lower in the ripe melting flesh cultivars (Nilo et al., 2012), while in the hard-melting flesh fruit ferredoxin increased in abundance during ripening (Jiang et al., 2020a,b). Subunit F of chloroplast NAD(P)H dehydrogenase (NDH) complex was more abundant in soft fruits. An increase in this complex would enhance electron transfer and generate ROS in chloroplasts or chromoplasts. In mitochondria, Nilo et al. (2012) found a decrease in a subunit of ATP synthase.

#### Oxidation-reduction ROS

Oxidative stress and an increase in reactive oxidative species (ROS) occur during fruit ripening. In the final phase of fruit ripening, accumulation of H_2_O_2_ and membrane lipid peroxidation occurs (Jimenez et al., 2002). Prinsi et al. (2011) found in both melting and non-melting peaches increase in peroxidase (POD) and superoxide dismutase (SOD), while catalase (CAT) decreased during ripening. SOD increased more in melting flesh fruits.

Chlorogenic acid (CHA) is a principal phenolic compound in nectarine fruit pulp and has antioxidant activity, which is positively correlated with ROS scavenging ability in peach and nectarine fruits. Xi et al. (2017) infiltrated nectarines with CHA at harvest and then held them at 25°C for 8 days. Peroxidase (POX) was reduced, while superoxide dismutase (SOD), glutathione reductase (GR), glutathione-S-transferase (GST), and monodehydroascorbate reductase (MHAR) were enhanced.

Zhang et al. (2011) found that hot water (48°C for 10 min) could activate the ascorbate-glutathione cycle. Treating fruits with hot water and 1-MCP, Jiang et al. (2014) found that ascorbate peroxidase (APX), dehydroascorbate reductase (DHAR) methionine sulfoxide reductase (MSR), glutathione peroxidase (GPX), and isoflavone reductase (IFR) were increased by the combined treatment. Lara et al. (2009) found a decrease in polyphenol oxidase (PPO) in heated peaches, which would inhibit internal browning.

Thioredoxins are small proteins, which are involved in cell redox regulation and were more abundant in ripe fruits than unripe fruits (Nilo et al., 2012). In the stony-hard and hard melting flesh cultivars glutaredoxin, a member of the thioredoxin family, was abundant (Jiang et al., 2020a). Three spots of eucaryotic translation initiation factor 5A (EIF5A) decreased in ripe fruits (Nilo et al., 2012). This protein facilitates protein synthesis by translocating mRNA from the nucleus to the cytoplasm. It has been linked to thermotolerance and oxidative stress resistance, and its decrease may enhance the oxidative stress associated with fruit ripening.

#### Stress proteins

Small heat shock proteins (sHSPs) act as chaperones to help prevent protein misfolding in stress situations. They are a large family, and the number of them increased during fruit ripening (Prinsi et al., 2011; Nilo et al., 2012). Heat-treated peaches with hot air for 3 days (Lara et al., 2009) or with hot water for 20 min (Jiang et al., 2014) induced sHSPs which remained present during shelf life. Peaches treated with hot water and 1-MCP, or with 1-MCP alone, had an increased presence of sHSPs as well as mitochondrial HSP70 and pathogenesis-related (PR) proteins (Zhang et al., 2012; Jiang et al., 2014). Treatment with chlorogenic acid to enhance oxidative reactions in nectarines led to an increase in sHSPs and a number of allergens many of which are also pathogenesis response (PR) proteins (Xi et al., 2017). In the hard-melting flesh peach, “Xiahui 8” sHSPs were induced at harvest (Jiang et al., 2020b), although chloroplast and mitochondrial sHSPs were less abundant in ripe fruits than in unripe fruits, a possible indication of senescence of the organelles (Jiang et al., 2020a). Dehydrins were also present in ripe fruits in a number of studies (Lara et al., 2009; Prinsi et al., 2011).

#### Secondary metabolism, lipids, and aroma

In the stony-hard and hard melting flesh peaches, chalcone synthase (CHS) the initial step in the flavonoid pathway, UDP-glucose-flavonoid 3-O-glucosyltransferase (UFGT) involved in stable anthocyanin synthesis, and dihydroflavonol 4-reductase (DFR) which catalyzes the reduction of dihydroflavonols to leucoanthocyanidins were upregulated in one or both fruits (Jiang et al., 2020a).

Leucoanthocyanidin dioxygenase (LDOX) involved in flavonoid biosynthesis increased in both melting flesh and non-melting flesh fruits, and was higher in the melting flesh (Prinsi et al., 2011).

Prinsi et al. (2011) found an increase in both cultivars, melting flesh and non-melting, in enoyl-coA hydratase (ECH) involved in β-oxidation of fatty acids. In the study of stony hard and hard melting flesh peaches, omega-6-fatty acid desaturase (FAD) and ECH were found in both cultivars but were higher in hard melting flesh fruits (Jiang et al., 2020a,b). Phospholipase D was found only in hard melting flesh fruits. Changes in membrane lipids occur during ripening which on the one hand leads to greater membrane leakage and on the other hand, the lipid breakdown products are involved in fruit aroma production.

### Proteomics of chilling injury

#### Segregating populations

A study using 2-DE was on an F2 population of the self-pollinating “Venus” nectarine which segregated according to their susceptibility to mealiness/wooliness in storage, among other traits (Almeida et al., 2016). The fruits were stored at 4°C for 21 days and then ripened to a similar softness before sampling. Of the 837 spots detected in fruit mesocarp protein extracts, 133 were identified as significant both by *t*-test and Wilcoxon statistical tests (Almeida et al., 2016). Identified proteins accumulated in juicy fruits in comparison to mealy/wooly fruits include response to stress proteins and proteins associated with hormone synthesis. These included SAM synthase, ACO, and an abscisic acid ripening protein, along with other proteins related to response to stress such as CAT, cell wall metabolism (PG), and transmembrane transport. In contrast, proteins that accumulated preferentially in mealy/wooly fruits included proteins related to oxidative stress such as glutathione-S-transferase (GST), thioredoxin reductase, and stress proteins such as HSPs, dehydrin, and LDOX. The authors suggest that the catalase in the juicy fruits may lead to a greater ability of these fruits to resist oxidative stress, in comparison to those that develop mealiness/wooliness. However, in a proteomic study of ripening, it was found that CAT decreases as ripening proceeds, while other redox enzymes increase (Prinsi et al., 2011). The mealy/wooly fruits are undergoing oxidative stress due to the CI and so have higher amounts of oxidative stress proteins.

The F2 population of “Venus” nectarines has been used in a genomic investigation of genes involved in traits associated with mealiness/wooliness and with maturity date (Nunez-Lillo et al., 2015). They found four co-localizing QTLs for mealiness/wooliness on LG4. Genes in this area included those for cell wall synthesis and ethylene production. Other genomic studies have found SAM synthase, PG, and LDOX localized to QTLs for either mealiness/wooliness or internal browning (Lurie, 2021).

Another proteomic study used “Venus” nectarines (though not a segregating F2 population) which were stored for 5 weeks at 5°C and 1 day at 20°C (Giraldo et al., 2012). All the fruits showed chilling injury, both internal browning and mealiness/wooliness. 2-DE gels of healthy unstored fruits and stored fruits were compared. While this study identified only a few proteins, among them were CAT and an sHSP (small HSP 1) in the healthy fruits, while the chilling injured fruits had stress proteins including PR proteins chitinase, thaumatin-like proteins, and glutathione peroxidase. Thaumatin-like proteins have been found abundant in peaches during storage in other studies (Dagar et al., 2010; Nilo et al., 2010). It has been suggested that this protein, which has the activity as an antifreeze protein may be involved in trying to protect against CI (Dagar et al., 2010). CAT was found in the juicy fruits in the study of the F2 population, and a decrease in CAT has also been proposed as a marker for CI (Nilo et al., 2010). The small HSP 1 protein has been linked to the stabilization of cell components under stresses such as heat and oxidative stress, and in tomato that has been heated before low-temperature storage, the small HSPs help protects against CI (Sabehat et al., 1998).

#### Other comparisons

Nilo et al. (2010) used DIGE to compare four post-harvest stages of “O'Henry” fruits; harvest, ripening 6 days at 20°C, 21 days at 4°C, and ripening after storage for 6 days at 20°C. The chilling injuries that manifested in the fruits after storage and ripening were mealiness/wooliness and internal browning. Their proteomic analysis identified 43 spots (18% of all the peptides present) with statistically different changes in the four conditions, and 39 could be identified. Of these seven were small HSPs, all of which were highest in ripe non-stored fruits along with PG and thioredoxin H. Small HSPs are a large family of protein chaperones and membrane stabilizers. Members of this family have been found in other studies to increase mealy peaches (Obenland et al., 2008). Thioredoxin H is important in cellular redox regulation (Gelhaye et al., 2004). Thioredoxin H and some of the small HSPs were absent in the chilling injured fruits after storage, while PG was present but in a smaller amount. In the fruits after storage, there were four proteins in high amounts; thaumatin-like protein, phosphoserine aminotransferase, α-1,4-glucan-protein synthase, and leucoanthocyanidin dioxygenase LDOX. Two dehydrins were present in stored and ripened after-storage fruits. LDOX has been suggested to be a potential marker for internal browning (Ogundiwin et al., 2008). CAT was present in fruits at harvest and low in fruits after shelf life, with or without storage, similar to Almeida et al. (2016) and Nilo et al. (2010).

A study of “Spring Lady” peach fruits examined the development of wooliness/mealiness in fruits stored at 0° for 24 days and then 5 days at 20°C. In this regime, many fruits developed mealiness/wooliness, but some became juicy. The two groups were compared using the gel-free system for separating proteins (Monti et al., 2019). In total, 1,015 proteins were identified in the peach fruit protein samples. From the 1,015 proteins, 227 were found to be differentially expressed between juicy and mealy at more than 2-fold. From these proteins, 165 were increased and 62 decreased in mealy/wooly fruits in comparison to the juicy fruits. The researchers interpret the higher number of proteins in CI-damaged fruits to indicate that wooliness/mealiness is linked to a reconfiguration of the proteome. In support of this, there was an increase in aminoacyl-tRNA synthesis proteins and a decrease in ubiquitylation and proteases. The former indicates active protein synthesis and the latter less turnover of proteins. In addition, DEPs involved in redox processes were reduced in mealy/wooly fruits, including SOD, glutathione peroxidase, APX, and NAD(P)-oxidoreductase. In the mealy fruits, 10 proteins involved in cell wall metabolism were higher than in juicy fruits, while only two were lower. The lower ones included PG, while the increased proteins in mealy fruits included PGI, PE, α-arabinofuranosidase, and β-xylosidase. The authors postulate that the difference in response of the fruits (some ripening normally and some developing mealiness/wooliness) is determined by the redox status of the fruits when it enters storage (Monti et al., 2019).

Yu et al. (2015) compared the storage of “Yulu” peaches stored for 30 days at 10°C or at 5°C. At the low temperature, the fruits developed internal browning, while at 10°C there was considerable decay. In the 2-DE study of protein differences, 79 proteins differed more than 3-fold between the two treatments, out of over 1,000 protein spots detected. Another study compared “Yulu” fruits stored at either 5°C or 0°C for 28 days (Wang et al., 2013). The fruits stored at 5°C developed internal browning during storage and the fruits stored at 0°C did not. At 0°C, there were lower levels of invertases, both acid and neutral, as well as sucrose synthase, and higher levels of sucrose phosphate synthase (SPS). Treating “Yulu” peach with heat (37°C air for 3 days) or methyl jasmonate (10 μmol/L for 24 h) prevented internal browning during storage at 5°C (Yu et al., 2016). The treatments also led to high levels of sucrose due to more SPS and lower acid invertase (AI), and an increase in phosphofructokinase (PFK) which maintained monosaccharide levels.

The stony-hard peach “Xiacui” peach was used in an iTRAQ and gel-free separation study to compare changes in the proteome during storage at 4°C for 15 and 30 days compared to 25°C at 7 and 14 days (Huan et al., 2019). The stony-hard peach is characterized by a lack of ethylene production and a firm flesh in mature fruits (Hayama et al., 2006). However, in this study, the firmness decreased from 24 N to 8 N after 14 days at 25°C or 30 days at 4°C. Other ripening measurements such as an increase in soluble solids content (SSC) and a decrease in titratable acidity (TA) occurred at 4°C, as they do in normal ripening. There was no report of CI symptoms in the fruits after low-temperature storage. Therefore, it is unclear how representative this study is of low-temperature storage leading to CI. However, there were some major differences found in the fruits at the two temperature regimes.

At each time point, there were almost equal DEPs that were higher or lower compared to the time of harvest, and the number of DEPs increased with the time of storage. A total of 325 DEPs were identified. Cell wall enzymes PG, PE, and β-gal increased in the low-temperature storage, while PL increased in the room temperature storage. Starch synthesis enzymes, glucose-1-phosphate adenylyltransferase (AGP), and starch phosphorylase (SP) were lower in cold stored fruits, while β-GC involved in starch degradation increased, as it did in normal ripening (Jiang et al., 2020a). Also, during low-temperature storage, Huan et al. (2019) found an increase in sucrose synthase and β-GC similar to normal peach ripening (Prinsi et al., 2011; Jiang et al., 2020a). Moreover, there was a suppression of glycolysis and pyruvate metabolism at low temperatures. Phosphofructokinase (PFK), triose phosphate isomerase (TPI), pyruvate kinase (PK), and pyruvate dehydrogenase (PDH) were decreased at low temperatures. At low temperatures, the changes in oxidative enzymes were similar to some changes reported for fruit ripening. There was an increase in four peroxidases (PODs) and a decrease in catalase (CAT) in fruits at 4°C, similar to the ripening of melting and non-melting peaches (Prinsi et al., 2011). In addition, there was an increase in two polyphenol oxidases (PPOs) and lipoxygenase (LOX). At 25°C storage, the only change in oxidative enzymes was a decrease in catalase (CAT) on day 7 and an increase in glutathione reductase (GR) and glutathione-S-transferase (GST) on day 14 (Huan et al., 2019).

Adaptation of plant tissue to low temperature increases membrane lipid unsaturation, which helps to prevent chilling injury, by maintaining the membranes in a liquid crystalline state (Lyons, 1973). The lipid components of the membranes change and membrane leakage increase if CI develops. In the low temperature stored fruits, stearoyl-ACP desaturase (SAD) which introduces a double bond was decreased and phospholipase C which aids in the degradation of lipids was increased, while no DEPs for lipid metabolism were observed in fruits held at room temperature.

Tanou et al. (2017) harvested “June Gold” peaches at commercial maturity and stored them immediately or pre-conditioned them at 20°C before 0°C storage. A third treatment allowed the fruits to remain on the tree until it was tree ripe. Pre-conditioned fruits had very little CI and higher ethylene production along with higher protein levels of the enzymes in the ethylene synthesis pathway, while the tree-ripe fruits had the highest CI (mealiness/wooliness, internal browning, and reddening). There were 171 DEPs between the treatments. During the period after storage, the pre-conditioned fruits had higher levels of proteins involved in energy, metabolism, defense, and signal transduction compared to fruits developing mealiness/wooliness. The energy metabolism proteins included triose phosphate isomerase (TPI), NADP-malic enzyme (NADP-ME), and glyceraldehyde-3-phosphate dehydrogenase (GAPDH). Proteins differently expressed between the treatments during CI development after 40 days at 0°C and 3 days at 20°C included phosphofructokinase 2 (PFK2) which indirectly regulates glycolysis and gluconeogenesis, pyruvate kinase (PK) the last step in glycolysis, glycogen phosphorylase (GP), subtilisin-like protease (SLP), and eukaryotic translation initiation factor (EIF). In addition, in the chilling injured fruits, there were higher levels of abscisic acid ripening protein (ASR) and leucoanthocyanidin dioxygenase (LDOX).

### Concluding remarks

Changes in the fruits' proteome during ripening are dynamic, mirroring the many processes that take place during this period; including an increase in respiration rate and ethylene production, changes in sugar and organic acid content, development of color pigments and volatiles, and changes in cell-walls and membranes, and production of ROS. All of these processes require multiple enzymes, along with translation factors and signaling proteins. Separating out what protein changes are involved in creating chilling injuries such as flesh wooliness/mealiness or flesh browning rather than normal ripening is a daunting process.

Table 1 details the treatments used in different proteomic studies to compare fruits with and without chilling injury. The fruits with chilling injury were all held at 4 or 5°C for varying times and then warmed up for ripening. The fruits with no chilling injury were either not stored or treated before storage to prevent chilling injury, or stored at a different temperature. Nonetheless, in many cases, the DEPs that were found in these varying studies overlap.

**Table 1:** Treatments used proteomic studies of peach or nectarine for prevention of chilling injury.

**Author**	**Cultivar**	**Treatment**	**Storage temperature and time**
Obenland et al. (2008)	O'Henry	None	1°C 3 wks then 23°C
Nilo et al. (2010)	O'Henry	Delayed storage	4°C 3 wks then 20°C
Almeida et al. (2016)	Venus	Segregating population	4°C 3 wks then 20°C
Giraldo et al. (2012)	Venus	No storage	4°C 3 wks then 20°
Zhang et al. (2010)	Hongtau	0°C	0° or 5°C 3 wks
Huan et al. (2019)	Xiacui	No storage	4°C 30 d
Tanou et al. (2017)	June Gold	Delayed storage	0° 40 d then 20°C

**Figure 2:**
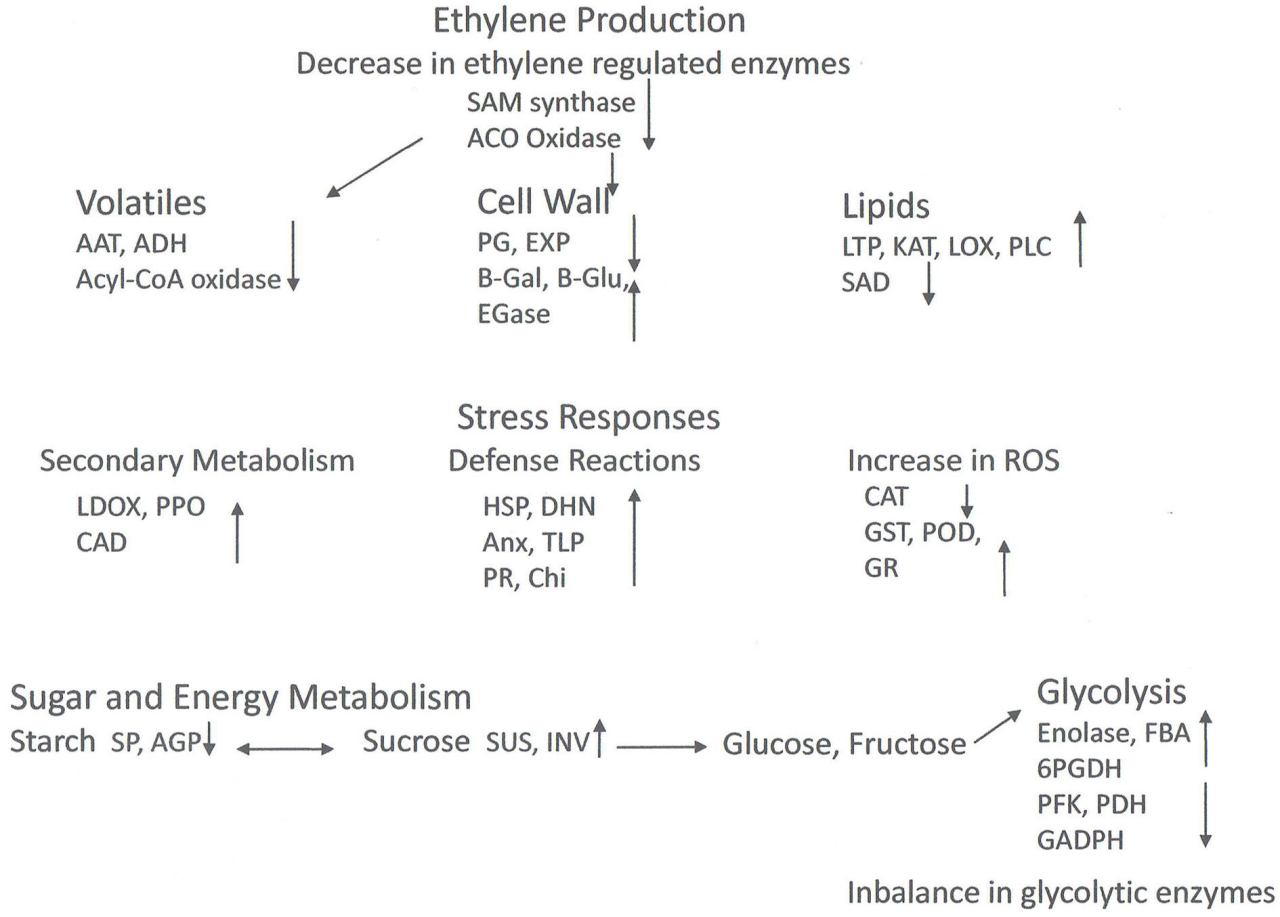
Model of proteomic changes in cellular processes that occur during chilling injury development in peaches and nectarines. Inhibition of ethylene synthesis in the cold affects processes including cell wall modulation and volatile production. The cold stress induces stress responses including phenol and flavonoid production, and biotic and abiotic stress compounds. Antioxidative enzymes are induced (except for catalase), but ROS accumulates nonetheless. Sucrose reserves decrease. Enzyme levels of glycolysis are both high and lower fruit without cold storage, suggesting an imbalance in activity. Abbreviations: AAT, Alcohol acyl transferase; ACO oxidase, 1-aminocyclopropane-1-carboxylic acid oxidase; ADH, alcohol dehydrogenase; AGP, glucose pyrophosphorylase; Anx, annexin; CAD, cinnamyl alcohol dehydrogenase; CAT, catalase; CHI, chitinase; DHN, dehydrin; EGase, endo-1,4-beta-D-glucanases; EXP, expansin; FBA, fructose bisphosphate aldolase; GADPH, glyceraldehyde 3 phosphate dehydrogenase; B-Gal, β-galactosidase; B-glu, β-glucanase; GR, glutathione reductase; GST, glutathione S-transferase; HSP, heat shock protein; INV, invertase; KAT, 3-ketoacyl-CoA-thiolase; LDOX, leucoanthocyanidin oxidase; LOX, lipoxygenase; LTP, lipid transfer protein; PFK, 6-phosphofructokinase; PDH, pyruvate dehydrogenase; PG, polygalacturonase; 6PGDH, 6-phosphogluconate dehydrogenase; PLC phospholipase C; POD, peroxidase; PPO, polyphenoloxidase; SAD, steroyl-ACP-desaturase; SAM oxidase, S-adenosylmethionine oxidase; SP, starch phosphorylase; SUS, sucrose synthase; TLP, thaumatin like protein.

Table 2 and Figure 2 show selected DEPs found in the different studies according to their functions. The enzymes of the ethylene pathway were decreased in chilling injured fruits in a number of studies, as was the ABA stress ripening protein (ASR). Ethylene production has been found to be decreased in wooly fruits (Zhou et al., 2001), and this would affect many ripening processes that are initiated by this hormone. Among these processes is cell wall softening due to polygalacturonase (PG), an enzyme induced by ethylene, and which is decreased in chilling injured fruits. Also, aroma development is induced by ethylene, and the DEPs of volatile production found in the studies were all decreased in chilling injured fruits. In contrast, a large number of stress-related proteins were increased in chilling injured fruits. This includes the enzymes to relieve oxidative stress except for catalase (CAT), which was higher in healthy fruits than in chilling injured fruits in three of the studies. A lipocalin was increased in one study. This is an enzyme in the xanthophyll pathway (Zhang et al., 2010). In addition to the antioxidant enzymes, there were increases in proteins related to both abiotic and biotic stress. Of the DEPs related to secondary metabolism which were identified in the various studies, all except isoflavone reductase-related proteins were higher in chilling injured fruits. Included in these are leucoanthocyanidin oxidase (LDOX) and polyphenol oxidase (PPO) which are involved in internal reddening and browning (Lurie, 2021).

**Table 2:** Proteomic changes during chilling injury development.

**Function**	**Change**	**Protein**	**Reference**
**Hormonal changes**			
	Decrease	Ethylene enzymes (SAM synthase, ACC oxidase)	Obenland et al., 2008; Nilo et al., 2010; Almeida et al., 2016; Tanou et al., 2017
	Decrease	ABA stress ripening protein (ASR)	Nilo et al., 2010; Almeida et al., 2016; Tanou et al., 2017; Huan et al., 2019
**Stress proteins**			
Oxidative stress	Increase	Glutathione S-transferase (GST)	Almeida et al., 2016
	Increase	Peroxidase (POD)	Huan et al., 2019
	Increase	Glutathione reductase (GR)	Giraldo et al., 2012
	Decrease	Catalase (CAT)	Nilo et al., 2010; Giraldo et al., 2012; Huan et al., 2019;
	Decrease	Temp. induced lipocalin	Zhang et al., 2010
Abiotic stress	Increase	Heat shock proteins (HSP)	Obenland et al., 2008; Zhang et al., 2010; Giraldo et al., 2012; Almeida et al., 2016
	Increase	Dehydrins (DHN)	Nilo et al., 2010; Zhang et al., 2010; Giraldo et al., 2012; Almeida et al., 2016
	Increase	Annexin (Anx)	Nilo et al., 2010; Giraldo et al., 2012; Almeida et al., 2016
	Increase	Thaumatin like protein (TLP)	Zhang et al., 2010
Biotic Stress	Increase	Pathogen related proteins (PR)	Zhang et al., 2010; Giraldo et al., 2012
	Increase	Chitinase (Chi)	Giraldo et al., 2012; Huan et al., 2019
**Secondary metabolism**			
	Increase	Leucoanthocyanidin oxidase (LDOX)	Nilo et al., 2010; Almeida et al., 2016; Tanou et al., 2017
	Increase	Polyphenoloxidase (PPO)	Zhang et al., 2010; Huan et al., 2019
	Increase	Cinnamyl alcohol dehydrogenase (CAD)	Zhang et al., 2010
	Increase	Hydroxymethybutenyl-4-diphosphate synthase (GCPE)	Obenland et al., 2008
	Increase	Isoflavone reductase related protein	Zhang et al., 2010
**Cell Wall and polysaccharide**			
	Increase	β-galactosidase (β-gal)	Huan et al., 2019
	Increase	Pectinmethylesterase (PME)	Nilo et al., 2010
	Increase	UDP-arabinopyroanose mutase (UAMP)	Nilo et al., 2010
	Increase	β-glucosidase (β-glu)	Huan et al., 2019
	Increase	Endoglucanase (EGase)	Huan et al., 2019
	Increase	Glucan-endo-1,3-β-glucosidase (GE-β-Glu)	Huan et al., 2019
	Increase	UDP-glucose pyrophosphorylase (UGlcPP)	Almeida et al., 2016
	Decrease	Glucose-1-phosphate adenylyltransferase (AGP)	Huan et al., 2019
	Decrease	Starch phosphorylase (SP)	Huan et al., 2019
	Decrease	Polygalacturonase (PG)	Nilo et al., 2010; Almeida et al., 2016
	Decrease	Expansin (EXP)	Obenland et al., 2003
**Glycolysis and gluconeogenesis**			
	Increase	Sucrose synthase (Sus)	Huan et al., 2019
	Increase	Enolase - 2-phosphoglycerate (2-PGA)	Zhang et al., 2010
	Increase	6-Phosphogluconate dehydrogenase (6PGDH)	Zhang et al., 2010
	Increase	Fructose-bisphosphate aldolase (FBA)	Tanou et al., 2017
	Decrease	6-Phosphofructokinase (PFK)	Tanou et al., 2017; Huan et al., 2019
	Decrease	Triosephosphate isomerase (TPI)	Tanou et al., 2017; Huan et al., 2019
	Decrease	Pyruvate kinase (PK)	Huan et al., 2019
	Decrease	Pyruvate dehydrogenase (PDH)	Huan et al., 2019
	Decrease	Glyceraldehyde 3-phosphate dehydrogenase (GAPDH)	Nilo et al., 2010; Tanou et al., 2017
	Decrease	Phosphoglycerate kinase (PGK)	Obenland et al., 2008
	Decrease	Phosphoenolpyruvate carboxykinase (PEPCK)	Tanou et al., 2017
**TCA pathway and amino acid synthesis**			
	Increase	NADP dependent isocitrate dehydrogenase (IDH)	Zhang et al., 2010
	Increase	NADP dependent malic enzyme (NAPD-ME)	Zhang et al., 2010
	Increase	Malate dehydrogenase (MDH) Alameida, Nilo	Nilo et al., 2010; Almeida et al., 2016
	Increase	Glutamate dehydrogenase (GDH)	Nilo et al., 2010; Zhang et al., 2010
	Increase	Phosphoserine aminotransferase (PSAT)	Nilo et al., 2010
	Increase	Chorismate mutase (CM)	Zhang et al., 2010
	Decrease	NADP-isocitrate dehydrogenase (NADP-ICDH)	Nilo et al., 2010
**Lipid metabolism**			
	Increase	Lipid transfer protein (LTP)	Zhang et al., 2010
	Increase	3-Ketoacyl-CoA thiolase (KAT)	Almeida et al., 2016
	Increase	Lipoxygenase (LOX)	Huan et al., 2019
	Increase	Phospholipase C (PLC)	Huan et al., 2019
	Decrease	Stearoyl-ACP desaturase (SAD)	Huan et al., 2019
**Membrane transport and membrane stability**			
	Increase	Dynamin	Tanou et al., 2017
	Decrease	Voltage dependent anion channel (VDAC)	Almeida et al., 2016
	Decrease	V-ATPase subunit A	Zhang et al., 2010
**Volatile production**			
	Decrease	Alcohol acyl transferase (AAT)	Huan et al., 2019
	Decrease	Alcohol dehydrogenase (ADH)	Huan et al., 2019
	Decrease	Acyl-CoA oxidase 2	Tanou et al., 2017

The cell wall modifying enzymes are involved in fruit softening, and the non-coordination of their activities is thought to lead to the wooly/mealy phenotype. Along with lower PG and EXP in chilling injured fruits were glucose-1-phosphate adenylyltransferase (AGP) a regulatory enzyme in glycogen synthesis, and starch phosphorylase (SP) which reversibly digests starch. However, there were a number of upregulated cell wall modifying enzymes including pectin methylesterase (PME) and enzymes which remove side chains from pectins and metabolize hemicellulose. The enzymes enhanced in the chilling injured fruits indicate that during chilling injury development there is extensive remodeling of the cell wall.

The enzymes involved in glycolysis or gluconeogenesis present a complicated picture. Four are higher in chilling injured fruits and seven are lower. In general, low-temperature storage will depress enzymatic activity as well as respiration, which is why fruits are held after harvest in the cold to decrease the rate of ripening and senescence. The finding that some enzymes are decreased and others increased in chilling injured fruits may indicate that normal metabolism is disrupted. Carbohydrate metabolism is important for both energy production and oxidoreduction processes, and the disruption of these processes can lead to physiological disorders.

In contrast, the enzymes involved in the tricarboxylic acid (TCA) and amino acid synthesis pathways are almost all higher in chilling injured fruits compared to healthy fruits. The higher level of these enzymes in chilling injured fruits indicates that the reduced power generated is important to try to counteract the oxidative damage occurring in the fruits' tissue. In addition, some of the enzymes, such as phosphoserine aminotransferase (PSAT), glutamate dehydrogenase (GDH), and chorismate mutase are involved in amino acid synthesis or breakdown. PSAT can lead to serine, which in turn can lead to glycine betaine (Nilo et al., 2010).

The lipid metabolism proteins found as DEPs in the studies were all higher in chilling injured fruits than in healthy fruits with the exception of stearoyl-ACP desaturase. Lipoxygenase (LOX), 3-ketoacyl-CoA thiolase (KAT), and phospholipid D (PLD) are all involved in the catabolism of membrane lipids. Membrane alteration and increase in membrane leakage are one of the major signs of chilling injury.

## Metabolomics

**Figure 3:**
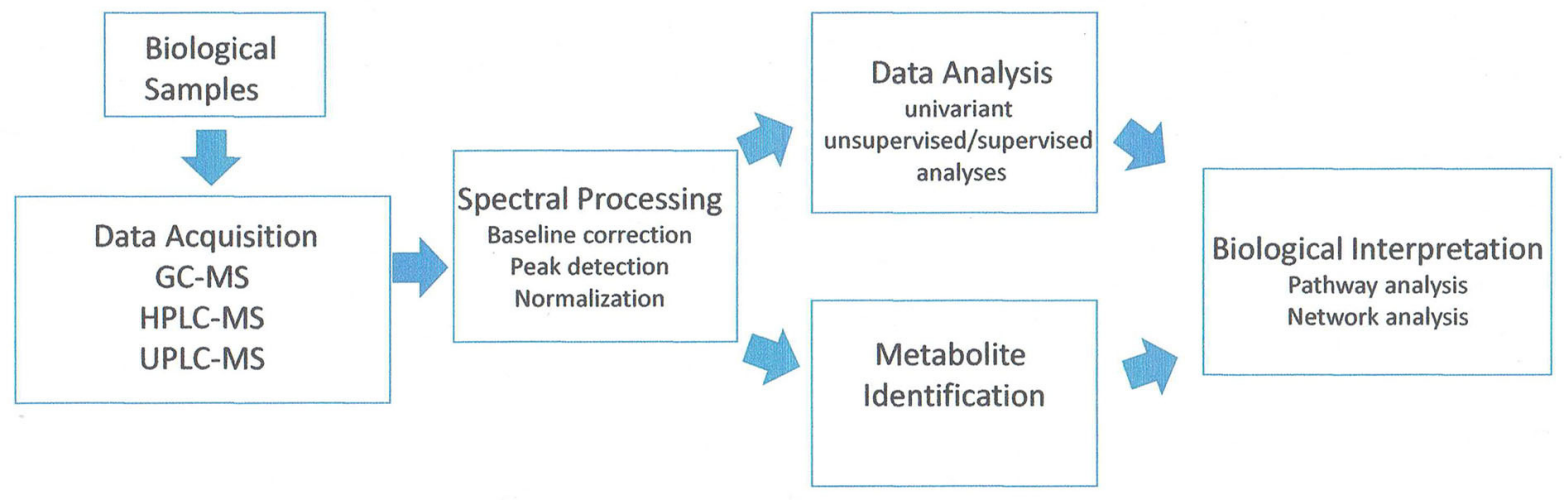
Analysis of workflow in untargeted metabolomic studies. Adapted from Alonso et al. (2015).

Metabolites are the intermediates or end products of multiple enzymatic reactions and therefore are the most informative proxies of the biochemical activity of the tissue. The current technologies allow the study of tens to hundreds of metabolites in complex biological samples (Patti et al., 2012). Analysis of the metabolic compounds in fruit cells can be conducted by a number of methods. Cellular contents are extracted generally using methanol–water for hydrophilic compounds. Hydrophobic compounds (such as lipids) are extracted in an organic solvent such as methanol: methyl tert-butyl ether: water (1:3:1) mixture (Giavalisco et al., 2011). Chromatographic separation techniques include liquid chromatography (either HPLC or UPLC) and gas chromatography (GS) coupled with mass spectroscopy (MS) systems (GC-MS, HPLC-MS, UPLC-MS). Most metabolic studies examining peach and nectarine have been done using GC-MS and derivatized samples. A few lipid studies were done with UPLC-MS. A flowchart of the method is shown in Figure 3.

### Sugars

Monti et al. (2019) examined polar metabolites to compare juicy or wooly/mealy “Spring Lady” fruits and found a decrease in soluble sugars in CI injured fruits. A drastic decrease was seen in both sugars and sugar alcohols (sucrose, fructose, glucose, ribose, xylose, galactose, sorbitol, and raffinose).

Pre-conditioned (48 h at 20°C before storage) “June Gold” peaches were resistant to CI (mealiness/wooliness, internal browning) and the fruits had higher concentrations before storage of several sugars compared to non-conditioned fruits (Tanou et al., 2017). These included both sugars (fucose, 1-O-methyl-alpha-D-glucopyranoside, maltose, and xylose) and sugar alcohols (maltitol). The fruits that developed CI after storage were lower in these compounds.

Lillo-Carmona et al. (2020) examined siblings from a nectarine segregating population (“Venus” x “Venus”) with contrasting levels of mealiness and juice content after cold storage. They compared fruits at harvest to fruits removed from 30 days 0°C storage and found that sugar, sugar acids, and hexose phosphate decreased markedly in fruits that would become mealy, compared to a very slight change in fruits that remained juicy.

Zhang et al. (2020) have correlated the level of ripeness of “Yulu” peaches at harvest as determined by sucrose content to susceptibility to chilling injury (in this case, internal browning). In another study of “Yulu” peaches held at 0 or 5°C, higher sucrose content in the 0°C stored fruits improved CI tolerance, while at 5°C internal browning developed and sucrose was low (Wang et al., 2013). Also, Yu et al. (2015, 2016, 2017) found that peaches with higher sucrose levels had greater chilling tolerance during cold storage. In one study (Yu et al., 2017), “Zajiao” and “Yulu” peaches were treated with 1-MCP to inhibit internal browning. 1-MCP slowed the degradation of sucrose, slowed the accumulation of glucose and fructose, and inhibited internal browning. He et al. (2018) stored “Yulu” and “Baifeng” peaches at four temperatures; 20, 10, 5, and 0°C. CI occurred mainly at 5°C and was accompanied by lower sucrose and higher reducing sugars. Sugars have been suggested to enhance the anti-oxidization system to eliminate free radicals, stabilize cell membrane structure, and act as osmoprotectants.

Metabolic profiling using GC-MS of “Dixieland” peaches hot air treated before storage was compared to cold stored fruits and to fruits ripened at 20°C (Lauxmann et al., 2014). The heat treatment has been found to prevent the onset of chilling injury in fruits subsequently held in cold storage (Lara et al., 2009; Bustamante et al., 2012; Lauxmann et al., 2012). In fruits ripened at 20°C, there was an increase in fucose and a decrease in glycerol and 1-methyl-glucoside. The sugars and sugar alcohols, particularly galactinol, were greatly affected by heat treatment. Other sugars which increased after heat treatment were raffinose, maltitol, fructose, glucose, and 1-O-methyl-glucoside as well as the disaccharides maltose, isomaltose, and trehalose. When fruits were placed in 0°C storage most (12 out of 17) sugars and alcohol sugars were not affected, but galactinol and raffinose increased and sucrose and glycerol decreased by 50%. A study that compared six peach cultivars with varying susceptibility to chilling injury (Bustamante et al., 2016) found that common to all were increases in galactinol and raffinose. However, the level of raffinose correlated with susceptibility to mealiness/wooliness. Xylose increase was found in the cultivar susceptible to chilling injury, indicating a change in the cell wall configuration.

Of the metabolites which might be involved in protecting from chilling injury, galactinol and raffinose showed the greatest increase in response to either heat or cold (Lauxmann et al., 2014). In other plant systems, these compounds have been found to be antioxidants or signals mediating stress responses.

In an experiment comparing three peach cultivars, “Red Haven” was found to be resistant to chilling injury and had higher levels of compounds with antioxidant activity, or acting as membrane stabilizers (Brizzolara et al., 2018). This cultivar had increases in sorbitol, maltitol, myoinositol, and sucrose. In all three cultivars raffinose, glucose-6-phosphate, fucose, xylose, and sorbitol increased during cold storage irrespective of their sensitivity to CI.

### Amino acids and organic acids

Monti et al. (2019) compared juicy fruits after storage to CI-damaged fruits and found a decrease in amino acids in wooly/mealy fruits, and no difference in organic acids. A drastic decrease was seen in all the amino acids detected (Ala, Asn, Gly, Glu, Ile, Ser, Thr, and Val). Pre-conditioned (48 h at 20°C before storage) “June Gold” peaches were resistant to CI (mealiness/wooliness, internal browning) and Val and Ile were found to be markers for tolerance to CI (Tanou et al., 2017). Pre-conditioned fruits that showed a CI-free phenotype had higher concentrations before storage of several amino acids (Asp, Glu, Ser, Tyr, Cys, Ile, Val, Thr, and Phe), and organic acids (glyceric acid and citric acid) than fruits that eventually expressed severe CI symptoms.

Lillo-Carmona et al. (2020) examined siblings from a nectarine segregating population (“Venus” x “Venus”) with contrasting levels of mealiness and juice content after cold storage. Amino acid levels were also lower in fruits that would develop a chilling injury. After cold storage, high relative amounts of amino acids such as Pro, Val, Ala, Ile, Ser, Leu, and Phe were present in the juicy phenotype, similar to pre-conditioned fruits without CI (Tanou et al., 2017).

Bustamante et al. (2016) examined similar and different changes in metabolites after cold storage in six cultivars with different susceptibilities to CI. Common changes were Asp, and Phe increase; and 2-oxo-glutarate and succinate decrease. Different changes were found in the sugars as noted above. Brizzolara et al. (2018) found that in three cultivars there were decreases in the organic acids citramalic, glucuronic, mucic, and shikimic during storage without regard to the sensitivity of the cultivar to CI.

Lauxmann et al. (2014) treated “Dixieland” peaches with hot air and then placed them at 0°C comparing them to fruits placed directly at 0°C or held at 20°C. The heated fruits were resistant to CI and the heat exposure affected organic acids, with citrate doubling its relative amount, and 2-oxo-glutarate, malate, and glycerate reduced in half their relative amount compared to fruits ripened at 20°C. Ten out of the 14 amino acids identified were modified upon heat exposure including Tyr, Asp, Ala, Gly, Phe, Pro, Glu, and Ser. Fruits stored directly did show changes in the main organic acids, but amino acids increased in 11 out of the 14 amino acids. It appears that temperature extremes (temperature stress) may lead to proteolysis and increases in amino acid content (Ferguson et al., 1994). Alternatively, Monti et al. (2019) found decreases in amino acids in wooly fruits after cold storage and suggest that this may be due to protein synthesis and/or amino acid oxidation (which also occurs during ripening). It appears that whether amino acids are found to increase or decrease during storage depends on the experimental conditions and the time of sampling.

### Lipids

Bustamante et al. (2018) examined lipidome remodeling in six peach cultivars with different susceptibilities to woolliness/mealiness and identified 59 lipid species. Storage at 0°C caused similar changes in all six cultivars which included decreases in monogalactosyldiacylglycerol (MGDG) species and increases in digalactosyldiacylglycerol (DGDG) and phosphatidylcholine (PC) species. However, DGDG increased more in resistant cultivars. The changes in the galactolipids (MGDG and DGDG) indicated changes in plastid membranes during storage with more stable membranes in the resistant cultivars. In the cultivars sensitive to CI, there was a decrease in PC(36:3) soon after cold storage began which did not occur in the resistant cultivars. The relative abundance of certain species of PC and phosphatidylethanolamine (PE) correlated with tolerance to CI. These changes showed the importance of the plasma membrane in CI tolerance.

Lillo-Carmona et al. (2020) took two siblings from a “Venus” x “Venus” cross with different propensities to develop woolliness/mealiness. They stored the fruits for 21 days at 0°C and examined juiciness after shelf life, but examined the fruits' lipids at the end of storage. At harvest, the fruits which would become juicy were high in diacylglycerol (DG), PC, PE, and phosphatidylglycerol (PG) species, while at the end of storage PC, PG, triacylglycerol (TAG), and DGDG species were increased and PE decreased. Conversely, at harvest, the fruits which would become mealy were rich in PG and MGDG species, while at the end of storage PG and MGDG species were decreased, while lysophosphatidylcholine (LPC) increased. These changes indicated remodeling in both plastid and plasma membranes.

Wang et al. (2017) held fruits at 8°C before 0°C (low-temperature conditioning) to prevent CI due to internal browning. They found that the treatment led to increases in phospholipid content and a preferential biosynthesis of the sphingolipid, glucosylceramide. They did not report on the lipids of the plastid membranes. Chen et al. (2021) treated stony-hard peaches with ethylene during storage to prevent internal browning, and also found increased levels of phospholipids, increased unsaturation of the acyl chains, and enhanced sphingolipid content.

### Other compounds

There are a number of compounds that do not fall into the above groups that different studies have indicated might have a role in preventing or enhancing chilling injury. Lauxmann et al. (2014) found that benzoate increased in cold stored fruits. Brizzolara et al. (2018) found that cold storage caused increases in gamma-aminobutyric acid (GABA), epicatechin, catechin, and putrescine in three peach cultivars. These are all compounds that were also affected by treatments to alleviate chilling injury as described below.

### Treatments to prevent chilling injury and effects on metabolites

Wang et al. (2021) used hot water to reduce CI (internal browning). They found higher levels of amino acids (Arg and Pro), and higher levels of phenolic compounds (chlorogenic acid, kaempferol, quercetin) and derivatives such as ɣ-aminobutyric acid (GABA) and polyamines in heated fruits. They suggest that these compounds contribute to enhancing antioxidant activity and alleviating membrane damage.

In addition to heat treatment, a number of other treatments have been tried to decrease chilling injury in stored peaches, and the studies examined the role of metabolites although not always by extensive metabolomics. The treatments included brassinosteroids (Gao et al., 2016), glycine betaine (Shan et al., 2016), melatonin (Cao et al., 2018a,b), nitric oxide (Shan et al., 2016), salicylic acid (Yang et al., 2020), and jasmonic acid (Chen et al., 2019) with or without heat treatment (Cao et al., 2010). Most of the studies examined white melting fleshed peach cultivars. The studies found metabolic compounds which helped prevent ROS development and maintain membrane stability.

Brassinosteroids are phytohormones involved in many plant developmental functions including responses to stress. Treating “Qinmi” peach fruits with 14-epibrassinolide before 1°C storage for 4 weeks reduced internal browning. The treatment reduced reactive oxygen species (ROS) and increased phenol and proline content in fruits (Gao et al., 2016). 14-Epibrassindolide was also found to inhibit the hydrolysis of phosphatidylcholine and phosphatidylinositol to phosphatidic acid and enhance unsaturated fatty acid contents (Hu et al., 2022). Glycine betaine, a quaternary ammonium alkaloid, is an osmotic adjustment compound that has a role in regulating stress responses. Exogenous glycine betaine treatment of “Yuhua No. 2” peaches decreased the severity of internal browning and mealiness/wooliness during 5 weeks at 0°C (Shan et al., 2016; Wang et al., 2019a,b). The treatment also increased the endogenous level of ɣ-aminobutyric acid (GABA) and proline, both compounds that protect membranes (Wang et al., 2019b). In addition, there were higher levels of unsaturated fatty acids in the phospholipids (Wang et al., 2019a). All these features led to better membrane function and a decrease in chilling injury. Similar results were found in treatments with nitric oxide, a compound that induces stress responses in peach and decreases CI. Treatment with nitric oxide induced GABA biosynthesis (Jiao and Duan, 2019), prevented ROS accumulation (Jiao et al., 2019) maintained high sucrose levels during storage (Han et al., 2018), and altered the lipid composition of fruits (Zhu and Zhou, 2006).

Melatonin is present in plants and is involved in growth, development, and stress responses. Treatment with melatonin induced hydrogen peroxide which activated the antioxidant systems and also increased ascorbic acid content (Cao et al., 2018a). After 28 days of storage at 4°C, treated “Hujing” peaches had very low levels of internal browning. Membrane lipid peroxidation was prevented and there was a higher ratio of unsaturated to saturated fatty acids (Gao et al., 2018). It also increased levels of polyamines, GABA, and proline (Cao et al., 2016). Cell wall disassembly proceeded normally in melatonin-treated fruits during and after low-temperature storage and prevented the development of mealiness/wooliness (Cao et al., 2018b).

## Concluding remarks

When fruits are stored at low temperatures, a series of protective mechanisms are triggered, which include the increase of cryoprotective molecules such as soluble sugars, sugar alcohols, and amino acids; modifications of the plasma membrane; the improvement of ROS scavenging activity; and the increase in stress proteins. Some fruits continue to respond to the cold temperature with these resistance processes, while others do so initially and then succumb to the stress and develop CI.

There appears to be a consensus that sugar metabolism plays a role in conferring resistance to CI. Sugars and sugar alcohols decreased in chilling injured fruits (Monti et al., 2019; Lillo-Carmona et al., 2020), while pre-conditioned fruits resistant to CI had higher sugars and sugar alcohols (Tanou et al., 2017). Zhang et al. (2020) correlated high sucrose levels at harvest with resistance to CI, and Wang et al. (2019b) found higher sucrose at 0°C storage than at 5°C again in correlation to chilling injury appearance. Treatments to induce CI resistance such as hot air, jasmonic acid or salicylic acid led to higher sucrose levels (Yu et al., 2016; Zhao et al., 2021a,b). Other sugars are also indicated in resistance to CI. Lauxmann et al. (2014) found increases in galactinol and raffinose in heated peaches, and Bustamante et al. (2016) correlated raffinose concentration as a candidate marker for chilling injury.

Amino acids decreased in chilling injured fruits, while pre-conditioned fruits had higher amino acid contents, and Tanou et al. (2017) found Val and Ile amino acids to be markers for tolerance to CI. Fruits at removal from storage that would become juicy had higher levels of amino acids compared to fruits that developed mealiness/wooliness (Lillo-Carmona et al., 2020). Proline which increases in cold stress was increased by treatments that protected against CI, such as heat treatment, glycine, and melatonin (Cao et al., 2016; Wang et al., 2019b).

In addition, to the metabolites that may play a role in membrane stability, such as sucrose, Pro, and polyamines, and which are found higher in fruits resistant to CI, there are changes in the membrane lipids. Bustamante et al. (2018) found changes in levels of galactolipids during 0°C indicating changes in plastid membranes. MGDG decreased during cold storage in all cultivars, while DGDG increased in the cultivars resistant to CI, changing the plastid membrane stability. In addition, in the plasma membrane components, the relative abundance of PE and PC species correlated with resistance to CI. Lillo-Carmona et al. (2020) also found that mealy fruits had a decrease in MGDG, and juicy fruits increased in DGDG. There were also differences between species of PE, PC, and PG between the juicy and mealy fruits and a large increase in saturated lysophosphatidylcholine (LPC) in CI-damaged fruits. With more studies on the lipidome of fruits during ripening and storage, the changes in the membrane lipids will be further resolved.

## Author contributions

The author confirms being the sole contributor of this work and has approved it for publication.

## Conflict of interest

The author declares that the research was conducted in the absence of any commercial or financial relationships that could be construed as a potential conflict of interest.

## Publisher's note

All claims expressed in this article are solely those of the authors and do not necessarily represent those of their affiliated organizations, or those of the publisher, the editors and the reviewers. Any product that may be evaluated in this article, or claim that may be made by its manufacturer, is not guaranteed or endorsed by the publisher.
